# Efficacious interventions for improving the transition readiness of adolescents and young adult patients with chronic illness: A narrative review of randomized control trials assessed with the transition readiness assessment questionnaire

**DOI:** 10.3389/fped.2022.983367

**Published:** 2022-09-28

**Authors:** Jiro Takeuchi, Yoshitoki Yanagimoto, Yuki Sato, Ryota Ochiai, Akinori Moriichi, Yuko Ishizaki, Takeo Nakayama

**Affiliations:** ^1^Department of Clinical Epidemiology, Hyogo Medical University, Nishinomiya, Japan; ^2^Department of Pediatrics, Kansai Medical University, Hirakata, Japan; ^3^Division of Specific Pediatric Chronic Disease Information, National Center for Child Health and Development, Tokyo, Japan; ^4^Department of Nursing, Graduate School of Medicine, Yokohama City University, Yokohama, Japan; ^5^Department of Pediatrics, Kansai Medical University Medical Center, Moriguchi, Japan; ^6^Department of Health Informatics, School of Public Health, Kyoto University, Kyoto, Japan

**Keywords:** adolescent, chronic disease, intervention, randomized controlled trial (RCT), review, transition readiness, questionnaires, young adult

## Abstract

**Objective:**

We inspected efficacious interventions to improve the transition readiness of adolescent and young adult patients with childhood-onset chronic illnesses using the Transition Readiness Assessment Questionnaire (TRAQ).

**Methods:**

Our narrative review was conducted on randomized control studies assessed with TRAQ for outcome measurement before and after the interventions. We included all patients with chronic diseases. We searched eight electronic database(s): Allied and Complementary Medicine Database (AMED) Allied and Complementary Medicine, BioSciences Information Service of Biological Abstracts (BIOSIS) Previews, Cumulative Index to Nursing and Allied Health Literature (CINAHL), the Cochrane Library, Embase, Ichu-shi, Medline, and Web of Science. The text words for the search of data sources were as follows: “(“transition readiness assessment questionnaire” OR TRAQ) AND 2011/01:2022/06[DP] AND (clinical AND trial OR clinical trials OR clinical trial OR random^*^ OR random allocation).” More studies were identified from the references in our reported study. This data set was independently cross-checked by two reviewers.

**Results:**

We identified 261 reports and collected three articles. The target diseases were type-1 diabetes, congenital heart disease, cystic fibrosis, and inflammatory bowel disease. All the studies excluded patients with intellectual disabilities. The age of the participants was distributed between 12 and 20 years. Nurse-provided web-based intervention of transition readiness was constructed using digital resources in two studies. The intervention ranged from 6 to 18 months. All the interventions were efficacious in improving transition readiness assessed with TRAQ scores, except for the self-advocacy score.

**Conclusions:**

We obtained three randomized control studies with TRAQ for outcome measurement. In two studies, web-based and nurse-led organized interventions were shown to improve transition readiness.

## Introduction

There is a growing concern about what medical care should be for adolescent and young adult patients with childhood-onset chronic diseases around the world. American Academy of Pediatrics, jointly with the American Academy of Family Physicians and American College of Physicians – American Society of Internal Medicine issued a Consensus Statement on Health Care Transitions for Young Adults with Special Health Care Needs in 1992 (1). While most young adults with special health care needs are able to become adults, many patients with severe medical conditions and disabilities — which limit their ability to function and result in complicated social, emotional, or behavioral sequelae — experience difficulty while transitioning from child to adult health care systems (1). Transition readiness is associated with independent self-care behaviors and patient quality of life, in addition to the appropriate shift to adult health care systems. The specific methodology for the transition is summarized in Six Core Elements (2). However, it has not been clarified as to what kind of interventions are effective in promoting self-care skills in adolescents and young adults with chronic diseases. In order to evaluate the efficacy of interventions aimed at transition to adult health care systems, it may be appropriate to examine the transition readiness status by interventions. At the moment, there are 10 kinds of tools to assess transition readiness. Among them, the Transition Readiness Assessment Questionnaire (TRAQ) (3) and TRxANSITION (4) have been verified for enough reliability and validity. Moreover, TRAQ, developed in 2011 (5, 6), has acquired internal validity, construct validity, and internal consistency (7). A higher TRAQ score indicates knowledge of the disease, skill, self-efficacy, positive outlook toward the future, and health-related quality of life (8–11). Conversely, a lower TRAQ score indicates non-adherence to drug therapy (12). In this study, we tried to review high-quality interventional research using the TRAQ for the outcome measurement to identify efficacious interventions and thus improve transition readiness for patients with childhood-onset chronic disease. We targeted randomized controlled studies for high-quality interventional research to avoid selection bias and confounding bias. The aspects to be addressed in this narrative review are as follows: participants, intervention, control, and outcome. We confirmed that increasing the score between the intervention group and control group allows for assessment of the quality of transition readiness. Therefore, employing efficacious interventions can improve the outcomes of the patients.

## Methods

### Study design

All randomized control trials assessed with the TRAQ before and after the intervention were included. Our narrative review was conducted by partially following the Preferred Reporting Items for Systematic Review and Meta-Analysis Protocols 2020 (PRISMA 2020) as a guide for the systematic review and meta-analysis protocol (13).

### Criteria for the included studies

We established eligibility criteria and exclusion criteria before the identification and selection of studies. The eligibility criteria are as follows: (1) research papers and not protocols or reviews and (2) studies assessed with TRAQ. The exclusion criteria are as follows: (1) non-intervention studies and (2) non-randomized studies.

### Patients

We included all patients who were diagnosed with childhood-onset chronic illness.

### Data sources and search strategy

On 18 June 2022, we searched eight electronic databases: Allied and Complementary Medicine Database (AMED) (14), BioSciences Information Service of Biological Abstracts (BIOSIS) Previews (15), Cumulative Index to Nursing and Allied Health Literature (CINAHL) (16), the Cochrane Library (17), Embase (18), Ichu-shi (in Japanese) (19), Medline (20), and Web of Science (21) for articles from 1st January 2011 to 30th June 2022. The text words used for the search were as follows: “(“transition readiness assessment questionnaire” OR TRAQ) AND (clinical AND trial OR clinical trials OR clinical trial OR random^*^ OR random allocation) AND 2011/01:2022/06[DP].” We checked the updates to all the databases through 30 June 2022. Additionally, more studies were identified from the references in our past reports. No limitation was imposed with regard to language. Publication type was limited to research papers of any length.

### Identification and selection of studies

First, we identified eligible studies through electronic searches and excluded duplicates. Second, we identified eligible studies and excluded duplicates of the same study by referring to the study title. Third, two reviewers (JT and YY) independently checked the reports at the title/abstract level and identified potentially relevant studies among the research assessed with TRAQ. Fourth, we assessed the studies and decided whether to include them based on the same eligibility criteria as the aspects of the randomized intervention. Any disagreements were resolved by an additional reviewer (YI).

### Data items and management

Characteristics of the studies, patients, interventions, and outcome measures were collected from each included study. Characteristics of the studies were established as columns in one table, and characteristics of the patients, interventions, and outcome measures were established as columns in another table.

One reviewer (JT) put the above data as variables into a data set in MS Excel. This data set was independently cross-checked by another reviewer (YY). They consulted with an additional reviewer (YI) regarding the variables with missing information. If we could not solve a problem, we employed expert opinion.

### Ethics

This narrative review does not require ethical approval. The data used here are neither individual nor private.

## Results

We searched eight database records identified (*n* = 261) from the following: no study from AMED, 90 studies from BIOSIS Previews, 4 studies from CINAHL, 92 studies from the Cochrane Library, 35 studies from Embase, 10 studies from Ichu-shi (Japanese), 15 studies from Medline, and 15 studies from Web of Science. After removing the duplicates, 147 studies were identified. After checking the reports at the title and abstract level, 47 studies were identified as potentially relevant. The excluded 100 studies were deemed to focus on other research themes. Of the remaining 47 studies, 11 studies were without an abstract or only included an abstract; 10 only included protocols; 3 were reviews; 10 included other questionnaires and not TRAQ; 5 were without intervention, and the last 6 were without randomization; therefore, we excluded these 45 studies. Finally, we included two randomized control studies (22, 23) in our review. An additional study (24) was identified from the references of our previous report (25). Overall, our study included three randomized control studies. The characteristics of the studies are presented in Table 1. All the reports are original articles in English. The collected data are presented in Table 2.

**Table 1:** Characteristics of studies.

**No**.	**Author(s)' name**	**Protocol**	**Clinical trial ID**	**Citation or publication**	**Year(s) of study**	**Year of publication**	**Location**	**Setting**	**Number of centers**	**Type of design**	**Sample size, *n***	**Observation period, months**
1	Huang et al. (24)	MD2Me - Texting to Promote Chronic Disease Management	ClinicalTrials.gov Identifier: NCT01253733	PEDIATRICS	October 2010 to March 2011	2014	United States	A tertiary care pediatric academic medical center	Single	A parallel randomized trial of two groups	80	16
2	Mackie et al. (22)	The CHAPTER II Study - Congenital Heart Adolescents Participating in Transition Evaluation Research	ClinicalTrials.gov Identifier: NCT01723332	Journal of the American College of Cardiology	2012 to 2016	2018	Canada	Outpatient clinics	Multiple (Not described in detail)	A parallel cluster randomized trial of two groups	Not described	30
3	Al Ksir et al. (23)	Motivational Interviewing to Improve Self-Management in Youth With Type 1 Diabetes	ClinicalTrials.gov Identifier: NCT04798937	Journal of Pediatric Nursing	2019 to 2020	2022	Tunisia	A pediatric endocrinology clinic	Single	A parallel randomized trial of two groups	60	6

**Table 2:** Characteristics of study patients, interventions, and outcome.

**No**.	**Type of basal disease**	**Exclusion criteria for participants**	**Number of participants at baseline, n**	**Gender distribution at baseline, n (%)**	**Mean (SD) or median [IQR], and range of age, years**	**Type of interventions**					**Controls**	**Type of outcome measure**	**Time(s) of outcome measurement**	**Withdraw during intervention periods, n (intervention group)**	**TRAQ score; means (SD) at baseline**	**TRAQ score; means (SD) after intervention**
						**Purpose**	**Interventionist**	**Contents**	**Tools**	**Amount**						
1	IBD, CF, T1D	Cognitive impairment	81 participants before intervention: CD 23; UC 11; CF 13; T1D 34)	Male 37 (45.7); 17 (42.5) in intervention group	17 between 12 and 20 in intervention group, 17 between 12 and 19 in control group IBD, 17 [16–18]; T1D, 17 [16–18]; CF 14 [13–16]	Disease management and skill-based interventions. Discussed about self-management constructs of monitoring disease symptoms.	Health care providers	Not face-to-face. Management program based on Bandura's Social Cognitive Theory.	Tailored short messages service and queries; 3–5 messages/ week. Reminder short messages service messages to reinforce previously introduced concepts and skills.	3–5 messages/ week for 1–2 months. Weekly after 2 months.	The control group: monthly messages via mail or e-mail addressing general health issues	Primary outcome: 1, disease status by using scales developed for each disease; the Pediatric Ulcerative Colitis Activity Index for patients with ulcerative colitis; the abbreviated Pediatric Crohn's Disease Activity Index for patients with Crohn disease;			
												the Cystic Fibrosis Clinical Score for patients with CF; the Diabetes Quality of Life Brief Clinical Inventory for patients with T1D 2, health status by using; the Karnofsky Performance Scale and the Pediatric Quality of Life Scale as quality of life	3 times (baseline, 2, and 8 months)	6 (2)	Overall score: 3.4 (0.9) in intervention group vs. 3.6 (0.7) in control group 2.9 (0.9) in CF group vs. 3.7 (0.8) in T1D group vs. 3.5 (0.7) in IBD group	Overall score: 3.5 (0.7) in intervention group vs. 3.8 (0.9) in control group, at 2 months; 4.0 (0.8) in intervention group vs. 3.8 (0.8) in control group, at 8 months*
												3, health literacy by using Test of Functional Health Literacy in Adults 4, readiness for transition and assesses performance of chronic disease self-management skills by using TRAQ scores (TRAQ 4.1) 5, managing one's own health and health care by using The Patient Activation Measure				
2	CHD	Less than a grade 6 level of reading or comprehension, and those with a heart transplant	125	Male 62 (51.2); 26 (44.8) in intervention group	16.9 (0.6) in intervention group, 17.1 (0.6) in control group, between 16 and 17	Session 1: inform participants about their heart condition. Session 2: motivate participants to self-manage and self-advocate.	One of two cardiology registered nurses	One-on-one sessions Session 1: creation of a MyHealth passport. Session 2: review of the education-related goal.	Available teleconference or video call in Session 2. Both sessions followed text message and or e-mail interaction. Below materials Session 1: a MyHealth passport. Session 2: 6 short videos, a video, 2 scenarios, a booklet, and a website.	Follow within 7 days Session 1: 1.0 h in a pediatric cardiology clinic visit. Session 2: 1.0-1.5 h for 2 months.	The usual care group: pertinent medical records were sent to adult CHD providers.	Primary outcome: excess time between pediatric and adult CHD care Secondary outcome: 1, change in the CHD knowledge (MyHeart) score 2, 1) change in TRAQ (20 items, version was not described) score; 2) Williams' self-management scale;	5 times (baseline, 1, 6, 12, and 18 months)	4 (3)	Self-management score: 2.9 (0.7) in intervention group vs. 2.9 (0.9) in control group Self-advocacy score: 4.0 (0.6) in intervention group vs. 3.9 (0.7) in control group	Self-management score; no numerical description^†^ Self-advocacy score; no numerical description^†^
												3) assessment of self-management via a cardiologist questionnaire 3, incidence of cardiac re-intervention				
3	T1D	A neurological disability (epilepsy, autism) or significant intellectual delay	66	Male 33 (50); 17 (51.5) in intervention group	15.3 (1.65) in intervention group, 15.06 (1.71) in control group, between 13 and 18	Development of general and disease self-management skills. To motivate the youths' engagement. Self-efficacy in changing his/her behavior.	A nurse	The individual; face-to-face sessions	Web-based videos and brochures. A MyHealth Passport. A calendar -tool.	20 min long with regular appointment with the pediatric endocrinologist. A 10 min follow-up call every month for the study period by nurse.	The control group: not described	Primary outcome: changes in TRAQ sores (TRAQ 4.1) Secondary outcome: change in HbA1c values	3 times (baseline, 3, and 6 months)	0	Overall score: 2.81 (0.86) in intervention group vs. 2.05 (0.57) in control group	Overall score: 3.53 (0.56) in intervention group vs. 2.11 (0.57) in control group, at 3 months; 4.25 (0.383) in intervention group vs. 2.31 (0.50) in control group, at 6 months

SD, standard deviation; IBD, inflammatory bowel disease; CF, cystic fibrosis; T1D, type 1 diabetes; CHD, congenital heart disease; HbA1c, Hemoglobin A1c. *Testing repeated-measures models testing the treatment × time interaction, including baseline (*p* = 0.02). ^†^Post-intervention TRAQ self-management scores and TRAQ self-advocacy scores were not listed, only illustrations testing mixed models (*p* = 0.03, and *p* = 0.67). ^‡^Testing with *t-*tests at 3 and 6 months (*p* ≤ 0.001, and *p* ≤ 0.001).

### Patients

The target diseases were type-1 diabetes in two studies, congenital heart disease in one study, cystic fibrosis in one study, and inflammatory bowel disease in one study. All the studies excluded intellectual disability. The subjects ranged in age from 12 to 20 years, with the mean or median age in each study ranging from 15 to 17 years.

### Intervention

In one study (24), the health care provider provided web-based and mobile phone-text-delivered disease management and skill-based interventions. The intervention was an 8-month technology-based disease management program based on Bandura's Social Cognitive Theory.

In the second study (22), nurses provided two nurse-led face-to-face sessions. The sessions comprised individualized 60-min educational sessions: Session 1 was created using the MyHealth Passport app (26). Session 2 reviewed the education-related goals including discussion, role-play, and reviews, with the same materials [short videos, video, scenarios, booklet (27), and website (28)], followed by a text message or e-mail interaction within 7 days.

In the third study (23), a nurse provided two face-to-face structured motivational interviews based on training manuals (29, 30). The sessions were conducted as 20-min face-to-face sessions and 10-min follow-up calls every month.

Contents of interventions were provided by digital resources in two studies. The length of the interventions ranged from 6 to 18 months in all studies.

### Control

Usual care was provided in the control groups in two studies; however, the remaining study did not describe the process followed.

### Outcome

The number of outcome types was five, five, and two in each study, respectively. All the outcomes were employed as a means to assess the TRAQ. The outcomes included disease status in all the studies. The outcomes of the two studies included health literacy or disease knowledge, which are not included in the TRAQ. All the interventions were efficacious at 6, 8, and 18 months, except for the self-advocacy score as evaluated by the TRAQ scores.

The mean TRAQ score at baseline was around 2.9 points, ranging from 2.05 to 3.7 in all three studies (22–24). Each chronic disease was shown in the same study (24), with a mean TRAQ score (SD) of 3.7 (0.8) points for patients with type-1 diabetes, 3.5 (0.7) points for patients with inflammatory bowel disease, and 2.9 (0.9) points for patients with cystic fibrosis.

The intervention in the overall TRAQ score showed a 0.6-point increase in the mean of the intervention group compared with a 0.2-point increase for the control group during 8 months for patients with inflammatory bowel disease, cystic fibrosis, and type-1 diabetes (24).

The intervention in the TRAQ self-management score did not have a numerical description but showed a significant increase in the graphic figure during 18 months among patients with congenital heart disease (23). The usual care in the TRAQ self-management score for patients with congenital heart disease did not have a numerical description but showed a significant increase in the graphic figure during 12 months; however, it did not show a significant increase in the graphic figure at 18 months (23). Neither the intervention nor usual care in the TRAQ self-advocacy score for patients with congenital heart diseases have a numerical description and did not show a significant increase in the graphic figure during 18 months (23).

The intervention in the overall TRAQ score showed an increase of 1.44 points in the mean of the intervention group compared with 0.26 points in that of the control group during 6 months among patients with type-1 diabetes (22).

## Discussion

The TRAQ is one of the best assessment tools (5), as it has cross-cultural validity and has thus been translated into many languages (25, 31–35). In our search results, three randomized control trial articles were assessed with the TRAQ. The developer of TRAQ recommends using the mean when it comes to a representative value. However, the authors of the study (24) instructed that acquiring four points or more as a TRAQ summary score can be regarded as starting to acquire the necessary disease management skills.

The target diseases were, of course, chronic illnesses, as the age for starting transition is related to the specific disease. In fact, the mean age in patients with type-1 diabetes was approximately 15 years, and that for patients with congenital heart disease was around 17 years. These differences mean that patients with a younger-onset disease tend to have a later starting transition than patients with an older-onset disease, as patients with a younger-onset disease are not adequately prepared for the transfer to adult care.

Before the discussion of interventions and assessment, we summarize the interventions and assessment, particularly related to the TRAQ for the three studies: In the first study, there was a 2-month intensive web-based and text-delivered disease management and skill-based intervention followed by a 6-month review period, with disease management and self-efficacy assessed with TRAQ (24). In the second, there were nurse-led face-to-face sessions in the intervention, with periods between the end of pediatrics and the beginning of adult medicine as the primary outcome, and change in the congenital heart disease knowledge in the TRAQ as the secondary outcome (22). In the third, 20-min face-to-face sessions were conducted as intervention, with changes in the TRAQ score as the primary outcome, and changes in hemoglobin A1c as the secondary outcome (23).

The intervention tools were applied with digital online devices for intervention staff to communicate with patients in two studies. A nurse was employed for intervention because nurses can work in both pediatrics and adult medicine. They additionally provide medical care for patients with chronic diseases. Nurses can improve the TRAQ scores of patients with nurses' independent support. On the other hand, medical social workers participate in connecting patients with social resources or the local society for transition readiness (2, 36, 37). We could not find a study in which a medical social worker led the intervention, and we hope such a randomized control trial study will be conducted with TRAQ in the future.

Patients with younger-onset disease tended to have lower mean TRAQ scores in the order of highest scores to lowest scores (24). These results indicated that patients with younger-onset diseases tend to have lower scores than patients with older-onset diseases. Sato et al. reported the TRAQ score (SD) for each chronic disease — 4.2 (0.6) points for patients with kidney disease, 3.3 (1.0) points for patients with congenital heart disease (25), and 4.2 (0.5) points for patients with other diseases (mainly rheumatoid disease) — assessed with the Japanese TRAQ. Thus, we should intensively intervene in patients with younger-onset diseases.

The intervention group showed a 0.6-point increase in the mean of the overall TRAQ score compared with the 0.2-point increase for the control group during 8 months among patients with inflammatory bowel disease, cystic fibrosis, and type-1 diabetes (24). The intervention group showed an increase of 1.44 points in the mean intervention group compared with 0.26 points in that of the control group during 8 months among patients with type-1 diabetes (22). After all, the longer the intervention was continued, the more efficacy was shown. In 2015, a Cochrane review reported that intervention made improvements in transition readiness, but it had low evidence (38). However, we collected novel evidence on transition readiness (22, 23).

Parental knowledge and parent-child discussions about transition are associated with higher TRAQ scores (9). Transition readiness requires intelligence. The three studies indicated some role of intelligence in the exclusion criteria. However, the TRAQ is one of the outcomes that assess transition readiness. However, the TRAQ has some limitations in terms of transition readiness. For instance, the TRAQ scores are not associated with appropriate consultation with medical experts for adults (3). Deliberateness would be required to increase appropriate consultations. Besides, while transitional intervention improves knowledge and transition readiness, it is unclear whether it improves the quality of life (39). We recommend the use of general quality of life scales as well as disease-specific scales for condition assessment (40–42). Disease-specified TRAQ can assess a disease-specific issue (43–45). The status or events of the disease can also be used to assess disease-specific issues from the current studies (22, 23). We require multiple assessments in practice (9, 46). Disease-specific evaluation tools are also recommended based on these results (24).

We try to provide patients with an opportunity to communicate with their guardians and health providers for their transition readiness through TRAQ. Such communication gives them an idea of how to deal with their disease.

In conclusion, both face-to-face and web-based interventions were shown to have the potential to improve transition readiness, as assessed by TRAQ. Nurses were considered to be key players in face-to-face interventions. All three studies intervened with the subjects repeatedly, suggesting that continuous support is efficacious.

## Author contributions

YS, YI, RO, and TN contributed to the conception of the study. JT substantially contributed to designing, searching databases, and drafting the article. JT, YY, AM, and YI contributed to the review of reports. JT, YY, YS, AM, YI, RO, and TN contributed to the critical revision of the article. All authors read and approved the final manuscript.

## Funding

JT received a grant for research from Japan Health Academy, the public interest foundation. YI received a grant for research activities from Chugai Pharmaceutical Co., Ltd.

## Conflict of interest

The authors declare that the research was conducted in the absence of any commercial or financial relationships that could be construed as a potential conflict of interest.

## Publisher's note

All claims expressed in this article are solely those of the authors and do not necessarily represent those of their affiliated organizations, or those of the publisher, the editors and the reviewers. Any product that may be evaluated in this article, or claim that may be made by its manufacturer, is not guaranteed or endorsed by the publisher.
